# SBRT von primären Nierenzellkarzinomen – Langzeitergebnisse der IROCK-Datenbank

**DOI:** 10.1007/s00066-023-02050-4

**Published:** 2023-02-06

**Authors:** Alexander Rühle, Simon Kirste

**Affiliations:** 1 Klinik für Strahlenheilkunde, Universitätsklinikum Freiburg, Robert-Koch-Str. 3, 79106 Freiburg, Deutschland; 2 Deutsches Krebsforschungszentrum (dkfz), Deutsches Konsortium für Translationale Krebsforschung (DKTK), Partnerstandort Freiburg, Freiburg, Deutschland

## Hintergrund und Ziel der Arbeit

Eine stereotaktische Radiotherapie (SBRT) ist eine nichtinvasive Behandlungsoption für Patienten mit primären Nierenzellkarzinomen, allerdings lagen bislang keine suffizienten Langzeitdaten vor.

## Patienten und Methoden

Diese Metaanalyse schloss Patienten ein, die eine SBRT eines primären Nierenzellkarzinoms zwischen 2007 und 2018 erhalten hatten, eine minimale Nachbeobachtungszeit von 2 Jahren aufwiesen, mindestens 18 Jahre alt waren und bei denen bisher keine Lokaltherapie des Nierenzellkarzinoms durchgeführt worden war. Fernmetastasen waren ein Ausschlussgrund. Insgesamt 190 Patienten von 12 Institutionen aus 5 Ländern (Australien, Kanada, Deutschland, Japan und USA) wurden in die Analyse eingeschlossen. Der primäre Endpunkt der Analyse war die Lokalrezidivrate gemäß RECIST-Kriterien.

## Ergebnisse

Bei 109 Patienten (57 %) wurde eine fraktionierte SBRT durchgeführt, die übrigen 81 Patienten (43 %) erhielten eine Einzeitbestrahlung. Im Median waren die Patienten 73,6 Jahre alt und hatten einen medianen Tumordurchmesser von 4,0 cm. Von den 128 Patienten mit verfügbaren Informationen zur Operabilität wurden 96 (75 %) als inoperabel eingeschätzt. Insgesamt 56 Patienten (29 %) hatten eine Einzelniere. Die mediane geschätzte glomeruläre Filtrationsrate (eGFR) betrug prätherapeutisch 60 ml/min pro 1,73 m^2^ und nahm um median 14,2 ml/min pro 1,73 m^2^ nach 5 Jahren ab. Sieben (4 %) Patienten wurden dialysepflichtig nach der SBRT. Die kumulative 5‑Jahres-Inzidenz lokaler Rezidive betrug 5,5 % nach einer medianen Nachbeobachtungszeit von 5 Jahren. Eine Einzeit-SBRT war mit einer geringeren Lokalrezidivrate im Vergleich zur fraktionierten SBRT assoziiert (*p* = 0,020). Insgesamt gab es lediglich bei einem Patienten höhergradige Toxizitäten, nämlich eine akute Grad-4-Toxizität (duodenales Ulkus) und eine chronische Grad-4-Toxizität (Gastritis).

## Schlussfolgerung der Autoren

Eine SBRT ist auch im Langzeitverlauf eine effektive und sichere Behandlungsoption für Patienten mit primären Nierenzellkarzinomen. Insgesamt unterstützen diese Daten den Einsatz einer SBRT bei Patienten mit primären Nierenzellkarzinomen, die inoperabel sind oder eine Operation ablehnen.

## Kommentar

Die Inzidenz primärer Nierenzellkarzinome ist ansteigend, unter anderem aufgrund der häufigeren Frequenz abdomineller Bildgebungen, welche zur vermehrten Detektion asymptomatischer Nierenläsionen führt [1]. Eine Nephrektomie ist der Goldstandard für Nierenzellkarzinome im Stadium I, wobei insbesondere im Stadium Ia eine nephronsparende Resektion bevorzugt werden sollte [2]. Ablative Techniken wie Radiofrequenzablation, Mikrowellenablation und Kryoablation sind Alternativen für Patienten, die inoperabel sind oder eine Operation ablehnen. *„Active surveillance“* ist eine Therapiemöglichkeit für Patienten mit sehr kleinen, nicht histologisch gesicherten Nierenläsionen (< 2 cm) aufgrund der häufig benignen Histologie in diesen Fällen.

Für Jahrzehnte spielte die Radiotherapie kaum eine Rolle in der Behandlung lokalisierter Nierenzellkarzinome, zum einen wegen der relativen Radioresistenz gegenüber normofraktionierter Bestrahlung und zum anderen wegen negativer Studienergebnisse in der neoadjuvanten und adjuvanten Therapiesituation [3]. Aufgrund des niedrigen α/β-Quotienten sowie anderer Apoptosemechanismen bei hohen Einzeldosen (ceramidvermittelte Apoptose) bestand jedoch eine radiobiologische Rationale zur Verwendung hoher Einzeldosen bei Nierenzellkarzinomen [4]. Die hohen Lokalkontrolldaten einer SBRT bei Nierenzellkarzinommetastasen sprachen ebenfalls dafür, eine SBRT bei primären Nierenzellkarzinomen zu untersuchen [5].

Eine zunehmende Anzahl retrospektiver und prospektiver Fallserien zeigte in den letzten Jahren die sehr hohen Lokalkontrollraten sowie geringen Toxizitäten dieser Behandlungsform bei primären Nierenzellkarzinomen [6], gerade auch in vulnerablen Kollektiven wie Patienten mit Einzelnieren [7], ≥ T1b-Tumoren [8] und Von-Hippel-Lindau-Syndrom [9]. Anders als eine Resektion ist die SBRT nicht invasiv und benötigt keine Narkose, was bei dem älteren und häufig multimorbiden Kollektiv relevant ist. Mit der SBRT können auch perihiläre Nierenzellkarzinome und größere Tumoren im Stadium T1b therapiert werden, wohingegen die thermalen Ablationsverfahren aufgrund beispielsweise des Risikos von Ureterstrikturen hier oftmals nicht eingesetzt werden können. In der aktuellen NCCN-Leitlinie wird die SBRT für inoperable Nierenzellkarzinompatienten im Stadium I als Option (Evidenzgrad: Kategorie 2B) genannt [2].

Die vorliegende Metaanalyse zeigt eine 5‑Jahres-Inzidenz lokaler Rezidive von lediglich 5,5 % nach einer medianen Nachbeobachtungszeit von 5 Jahren. Dies ist umso bemerkenswerter angesichts der Tatsache, dass relativ große Tumoren behandelt wurden und die Mehrheit der Patienten inoperabel war. Auch das krebsspezifische Überleben nach 5 Jahren war mit 92,0 % sehr hoch. Das progressionsfreie Überleben betrug 63,6 % nach 5 Jahren, wobei mehr als drei Viertel (*n* = 51, 77 %) der Todesfälle nicht tumorbedingt waren. Die insgesamt 66 Todesfälle verdeutlichen das fortgeschrittene Alter des Kollektivs sowie die bei Nierenzellkarzinompatienten häufiger vorhandenen internistischen Komorbiditäten wie arterielle Hypertonie, Adipositas und chronische Niereninsuffizienz.

Die nach 5 Jahren im Median um 14,2 ml/min pro 1,73 m^2^ abgefallene eGFR muss unter Berücksichtigung des Patientenkollektivs betrachtet werden: 49 % der Patienten hatten prätherapeutisch eine chronische Niereninsuffizienz von Grad ≥ 3 (GFR < 60 ml/min pro 1,73 m^2^) und 29 % eine Einzelniere. Die sieben Patienten (4 %), die während der Nachbeobachtungszeit dialysepflichtig wurden, hatten prätherapeutisch eine mediane eGFR von 33 ml/min pro 1,73 m^2^ und jeder dieser Patienten eine chronische Niereninsuffizienz von Grad ≥ 3.

Das Toxizitätsprofil der SBRT war sehr gering in dieser Metaanalyse: Bei 70 Patienten (37 %) kam es zu Toxizitäten von Grad 1–2, am häufigsten davon Fatigue (*n* = 51, 27 %). Es gab keine Grad-3-Toxizitäten und keine therapieassoziierten Todesfälle. Ein Patient entwickelte eine akute Grad-4-Toxizität (duodenales Ulkus) und zudem eine chronische Grad-4-Toxizität (Gastritis). Dieser Patient wurde mit 48 Gy in 4 Fraktionen behandelt und erhielt eine maximale Punktdosis von 54 Gy auf den Dünndarm. Zum Zeitpunkt der letzten Nachsorge (8,8 Jahre nach SBRT) war dieser Patient jedoch weiterhin am Leben und krankheitsfrei.

Eine explorative Analyse zeigte, dass eine Einzeit-SBRT mit einer geringeren Lokalrezidivrate und einem höheren progressionsfreien Überleben im Vergleich zur fraktionierten SBRT assoziiert war, während sich die Toxizitätsrate nicht unterschied. Auch in der multivariaten Analyse blieb die fraktionierte SBRT mit einer höheren Rate an Lokalrezidiven verglichen mit der Einzeit-SBRT assoziiert (*p* = 0,04). Zum aktuellen Zeitpunkt muss diese Beobachtung jedoch mit Vorsicht interpretiert und in weiteren Studien untersucht werden.

An Limitationen der hier diskutierten Studie sind unter anderem der Einschluss auch retrospektiver Patientendaten und die nicht obligate histologische Sicherung vor SBRT zu nennen (histologische Sicherung bei 83 %). Zudem waren das Patientenkollektiv und die SBRT-Technik heterogen, was aber die tatsächlichen aktuellen Behandlungsmuster weltweit widerspiegelt.

Zusammengefasst erhöht diese hochrangig veröffentlichte Studie aufgrund der nun verfügbaren Langzeitdaten zur Effektivität und zum Nebenwirkungsprofil die Evidenz für den Einsatz einer SBRT bei primären Nierenzellkarzinompatienten im Stadium I, welche nicht operabel sind oder eine Operation ablehnen. Zur Verbesserung des Evidenzgrads einer SBRT bei primären Nierenzellkarzinomen sind weitere prospektive (randomisierte) Studien nötig. Beispiele hierfür sind die RADSTER-Studie (NCT03811665), bei der zwischen einer Einzeit-SBRT (1 × 25 Gy) und einer Radiofrequenzablation randomisiert wird, oder die multizentrische FASTRACK-II-Studie (NCT02613819), bei der Tumore ≤ 4 cm mit einer Einzeit-SBRT (1 × 26 Gy) behandelt werden und Tumore > 4 cm mit einer fraktionierten SBRT (3 × 14 Gy).

*Alexander Rühle und Simon Kirste, Freiburg*

## References

[1] Capitanio U, Bensalah K, Bex A, Boorjian SA, Bray F, Coleman J (2019). Epidemiology of renal cell carcinoma. Eur Urol.

[2] National Comprehensive Cancer Network (2023) Kidney cancer (version 3.2023). https://www.nccn.org/professionals/physician_gls/pdf/kidney.pdf. Zugegriffen: 7. Jan. 2023

[3] Rühle A, Andratschke N, Siva S, Guckenberger M (2019). Is there a role for stereotactic radiotherapy in the treatment of renal cell carcinoma?. Clin Transl Radiat Oncol.

[4] Siva S, Kothari G, Muacevic A, Louie AV, Slotman BJ, Teh BS (2017). Radiotherapy for renal cell carcinoma: renaissance of an overlooked approach. Nat Rev Urol.

[5] Hoerner-Rieber J, Duma M, Blanck O, Hildebrandt G, Wittig A, Lohaus F (2017). Stereotactic body radiotherapy (SBRT) for pulmonary metastases from renal cell carcinoma—a multicenter analysis of the German working group “stereotactic radiotherapy”. J Thorac Dis.

[6] Correa RJM, Louie AV, Zaorsky NG, Lehrer EJ, Ellis R, Ponsky L (2019). The emerging role of stereotactic ablative radiotherapy for primary renal cell carcinoma: a systematic review and meta-analysis. Eur Urol Focus.

[7] Correa RJM, Louie AV, Staehler M, Warner A, Gandhidasan S, Ponsky L (2019). Stereotactic radiotherapy as a treatment option for renal tumors in the solitary kidney: a multicenter analysis from the IROCK. J Urol.

[8] Siva S, Correa RJM, Warner A, Staehler M, Ellis RJ, Ponsky L (2020). Stereotactic ablative radiotherapy for ≥T1b primary renal cell carcinoma: a report from the international radiosurgery oncology consortium for kidney (IROCK). Int J Radiat Oncol Biol Phys.

[9] Kirste S, Rühle A, Zschiedrich S, Schultze-Seemann W, Jilg CA, Neumann-Haefelin E (2022). Stereotactic body radiotherapy for renal cell carcinoma in patients with von Hippel-Lindau disease-results of a prospective trial. Cancers (Basel).

